# Genetic influences of autism candidate genes on circuit wiring and olfactory decoding

**DOI:** 10.1007/s00441-020-03390-8

**Published:** 2021-01-30

**Authors:** Renée Hartig, David Wolf, Michael J. Schmeisser, Wolfgang Kelsch

**Affiliations:** 1 Department of Psychiatry & Psychotherapy, University Medical Center, Johannes Gutenberg-University, 55131 Mainz, Germany; 2 Focus Program Translational Neurosciences (FTN), University Medical Center, Johannes Gutenberg-University, 55131 Mainz, Germany; 3 Central Institute of Mental Health, Medical Faculty Mannheim, Heidelberg University, 68159 Mannheim, Germany; 4 Institute for Microscopic Anatomy and Neurobiology, University Medical Center, Johannes Gutenberg-University, 55131 Mainz, Germany

**Keywords:** Behavior, Mice, Olfaction, Social, Shank2, Synaptic wiring

## Abstract

Olfaction supports a multitude of behaviors vital for social communication and interactions between conspecifics. Intact sensory processing is contingent upon proper circuit wiring. Disturbances in genetic factors controlling circuit assembly and synaptic wiring can lead to neurodevelopmental disorders, such as autism spectrum disorder (ASD), where impaired social interactions and communication are core symptoms. The variability in behavioral phenotype expression is also contingent upon the role environmental factors play in defining genetic expression. Considering the prevailing clinical diagnosis of ASD, research on therapeutic targets for autism is essential. Behavioral impairments may be identified along a range of increasingly complex social tasks. Hence, the assessment of social behavior and communication is progressing towards more ethologically relevant tasks. Garnering a more accurate understanding of social processing deficits in the sensory domain may greatly contribute to the development of therapeutic targets. With that framework, studies have found a viable link between social behaviors, circuit wiring, and altered neuronal coding related to the processing of salient social stimuli. Here, the relationship between social odor processing in rodents and humans is examined in the context of health and ASD, with special consideration for how genetic expression and neuronal connectivity may regulate behavioral phenotypes.

## Introduction

Olfaction is important for many species, with a strongly conserved evolutionary basis (Heymann 2006; Auer et al. 2020) and far-reaching effects on the ongoing neuronal processes related to a range of vital functions expressed by chemosensory beings. The decoding of olfactory information is ever more essential to understanding its influence on behavior, in guiding social interactions, and in learning and memory. Invaluable insight has been drawn from the use of murine and invertebrate models, for instance, to study the underlying molecular and cellular processes, often using methodological techniques not feasible in humans. Transgenic animals that model human disorders have great potential in vital scientific research and in developing applicable treatments.

One disorder that bears prevalence in humans is autism spectrum disorder (ASD), whereby a range of behavioral deficits in the realm of social interaction and communication is evident (Muhle et al. 2018). This neurodevelopmental disorder manifests early in childhood and is often accompanied by repetitive and/or stereotypical behaviors (American Psychiatric Association 2013). In the USA, an estimated 1-in-54 children and adolescents were diagnosed with ASD between 2014 and 2016 (Xu et al. 2018). The numbers are particularly striking as the calculated prevalence at the turn of the twenty-first century was approximately 1-in-150 (Autism and Developmental Disabilities Monitoring Network Surveillance Year 2002 Principal Investigators), indicating a gradual increase over time. While the proportion of ASD diagnoses may plateau, as potentially indicated by a rather steady percentage between the 2014 and 2016 period, it is unknown if clinical expertise and diagnostics have truly reached a threshold for stemming the decades-long increase in ASD cases.

Given that a plethora of genetic and environmental risk factors have been identified, pointing to a complex, polygenic etiology of ASD (Sung et al. 2005; Jansen et al. 2020), it is to no surprise that the causes of ASD are not fully understood. The extent to which mutated genetic expression is influenced by environmental factors and the effects on neuronal assemblies is discussed here. Furthermore, the role of olfactory decoding is examined, especially within the context of social information processing, and its effects in ASD, largely considering the genetic links to synapse formation and neuronal wiring.

In particular, the impaired social behavior and communication in patients with ASD is not an isolated higher-order dysfunction, but rather a broad dysfunction of many brain circuits entailing both impaired primary sensory processing and perception (Robertson and Baron-Cohen 2017). Sensory deficits in ASD patients have been reported to occur early in development (Kern et al. 2006) and appear to predict clinical outcomes later in life (Kaldy et al. 2011; Gliga et al. 2015). It is, therefore, promising to study the sensory processing of social information in healthy individuals, patients, and in ASD models at various levels, whereby the underlying genetic basis and convergence of behavioral and physiological phenotypes can be identified.

The importance of olfaction for social cognition and its impairment in ASD is highlighted in this article, largely drawing from insight gathered using murine models and from clinical cases. The genetic determination of olfactory circuits and the influence of ASD candidate genes on social odor processing and olfactory decoding is also discussed. Notably, this is not a comprehensive review covering all the literature on the topics, but rather a focused depiction of the relationships as illustrated by exemplary studies, noting the current progress in the field, and where future studies may progress our understanding of social olfaction within the context of supporting healthy function and behavior.

## Olfaction in social cognition and ASD

**Fig. 1:**
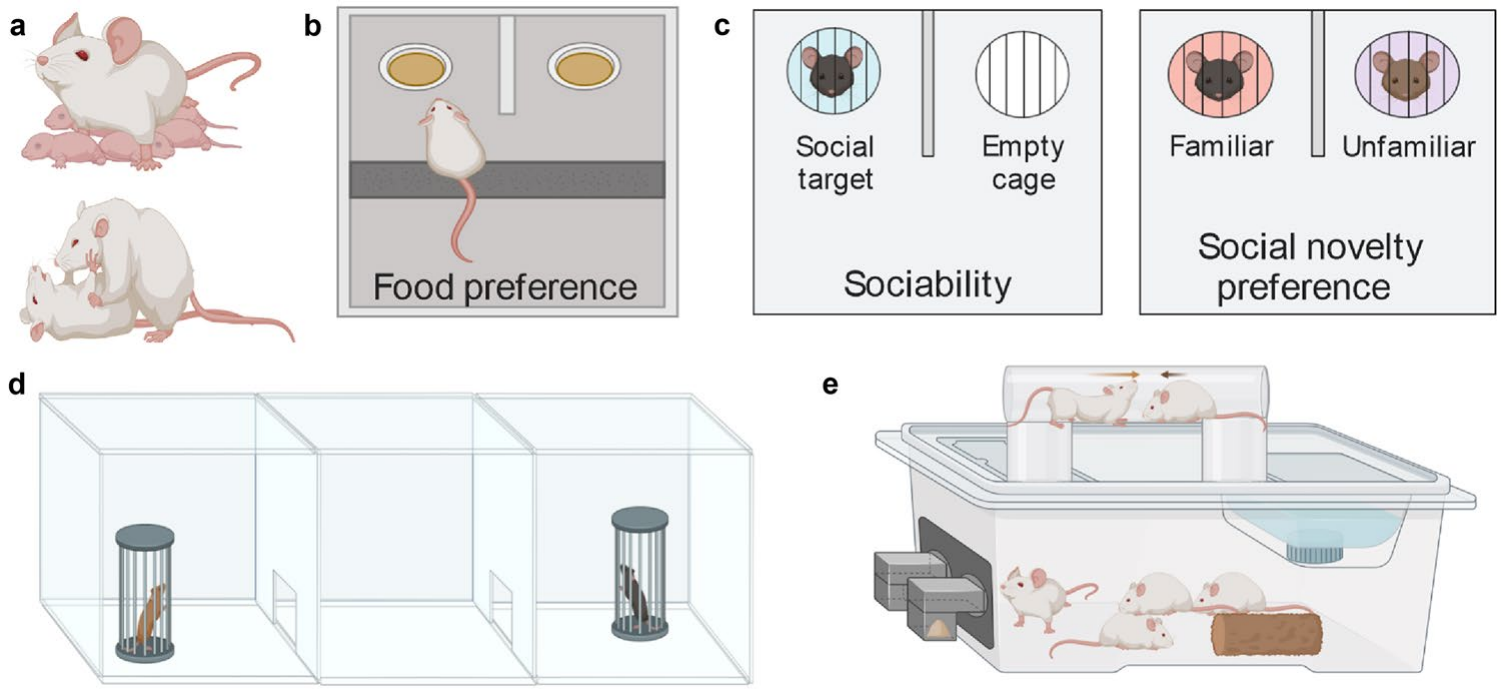
Social behavior assessment in murine models. Social interactions can be studied using behavioral assays, where the behaviors observed in different settings help inform the role of social cues. Such assays aid in isolating specific sensory components from an interaction experience. Social behaviors include interactions with **a** offspring, facilitating maternal behavior (top), such as pup retrieval, and from conspecifics which can elicit aggressive behaviors (bottom). The **b** food preference test and **c** sociability (left) and social novelty preference (right) tests can also be implemented in a **d** 3-chamber testing arena where social cues can be positioned at each end of a linearly oriented row of chambers, consisting for instance, of a neutral middle zone where test animals make a decision where to spend most time exerting exploratory behavior. **e** A housing environment where odor and water ports have been integrated to run conditioning paradigms and examine group behavior (e.g., social hierarchies). Figure utilized BioRender for standard graphical illustrations

Olfactory stimuli influence a myriad of social behaviors. This is, in part, because olfactory stimuli produced by conspecifics carry information relevant for behavior and social cognition. Olfaction-dependent social behaviors include mating, aggression, and social recognition (Liberles 2014; see also Fig. 1). Such considerations of ethology may provide additional opportunities to study functional impairments along a range of social tasks.

Traditionally, many different behavioral assays have been designed and validated for specific domains of social behavior. To name only a few which are widely used for the characterization of social behaviors, there is the 3-chamber social interaction test for sociability (Fig. 1c, left; Fig. 1d), social transmission of food preference (Fig. 1b), and the partner preference test, or social novelty preference (Fig. 1c, right), for social recognition and memory. While behavior demonstrated by social task performance is greatly influenced by olfaction, it should also be acknowledged that sensory input from other modalities, such as vision and audition, can also play a role in modulating behavior. By design these tests examine a limited subset of naturally occurring social behaviors, and the exact role of olfactory processing remains unknown and critical care should be taken to control for the influence of other modalities of sensory input.

Rodents actively explore their environment in an effort to sample information and process relevant social cues. Social odors are mixtures of volatile and non-volatile molecules produced by a conspecific and signal individuality or elicit a certain behavioral response. Primary sources for these are exocrine glands in the facial area, sweat, urine, and feces. Nevertheless, odors can carry social information even if they are not directly emitted or produced by an interaction partner but are associated with a social value (e.g., the perfume of your partner). With this, odor association and sampling context can further influence action and modulate behavior.

While much attention has been paid to perceptual sensory deficits in tactile sensitivity, auditory filtering, and in the visual domain (e.g., misinterpretation of facial expressions or body language) (Thye et al. 2018), there is increasing evidence that chemo-senses, such as taste and smell, are impaired in ASD (Bennetto et al. 2007; Boudjarane et al. 2017; Barros and Soares 2020). Children with ASD are often described as “picky eaters” by their parents and studies show, indeed, that food selectivity is narrower than in typically developing children (Williams et al. 2005; Schreck and Williams 2006; Cermak et al. 2010). This phenomenon is often attributed to greater sensory sensitivity to food texture, but smell plays a crucial role (Cermak et al. 2010).

Several studies have investigated and characterized olfactory function in ASD patients, but reveal partially divergent results in terms of how sensitivity to odor identification and enhanced perception is compared with typically-developed individuals (Bennetto et al. 2007; Galle et al. 2013; Addo et al. 2017; Muratori et al. 2017). Two meta-analyses that looked at odor detection thresholds found large heterogeneity in effect sizes and offered no clear conclusion whether there is hyper- or hypo-sensitivity in the olfactory system across the spectrum of ASD patients (Tonacci et al. 2017; Larsson et al. 2017). One of these meta-analyses, however, found more consistent results for impaired odor identification (Tonacci et al. 2017). All in all, any divergence across these studies might relate to the different etiological roots of autism in individual cases across the studied population. Further studies are needed to more reliably determine the olfactory impairments in ASD (Larsson et al. 2017). Also, many studies rely on active odor reporting from participants, which could be unreliable in cases with impairment of communication, or with young patients.

A more promising read-out for olfactory processing is the sniff response to odors, especially in clinical populations with impaired communication and with subliminal odor presentation. Sniff pattern and its modulatory change are more than just the subject’s breathing rhythm. Salient odors evoking emotional responses have been found to modulate olfactory cortices as well as breathing rhythmicity (Krusemark et al. 2013). Notably, breathing and olfaction are highly coupled functional processes (Perl et al. 2019). In a study that non-invasively monitored sniffing in socially interacting rats, sniff behavior conveyed information about the hierarchy and social status between two interaction partners (Wesson 2013).

Humans actively (often subconsciously) sample chemosensory cues from the social domain. For example, after shaking hands with a stranger from the same sex humans sniff their hand significantly more, but after shaking hands with someone from the opposite sex, only significantly sniff the hand that did not touch the stranger (Frumin et al. 2015). Taking advantage of these features of the sniff response, a study investigated the response to pleasant and unpleasant odors in typically-developing and autistic children (Rozenkrantz et al. 2015), finding that healthy children had a stronger decrease in normalized sniff volume after presentation of an unpleasant odor as compared with a pleasant odor. Children with ASD, however, showed no significant dissociation of sniff response to the two odors, and the severity of autistic symptoms was correlated to aberrant sniffing (Rozenkrantz et al. 2015). Since perceptual impairments in ASD in visual and other sensory domains are influenced by social cues, it is of great interest how the processing of social chemosensory signals is impaired in patients.

In regard to hand sniffing after greeting a person, autistic adults exhibited social odor sampling behavior equal to healthy controls (Endevelt-Shapira et al. 2018). Autistic males were also equally capable of discriminating body odors (i.e., sweat collected from skydivers or men walking) and rated them comparably to healthy controls in pleasantness, intensity, and fearfulness. However, when presented with either “fear sweat” from the skydiver or control sweat (both below perceptual threshold), healthy adults exhibited greater autonomic arousal (by electrodermal activity) to fear sweat than to control sweat, while ASD patients showed no change in the autonomic response (Endevelt-Shapira et al. 2018). The opposing behavioral response between ASD participants and healthy participants to social chemosensory cues also reoccurred in an implicit measure of trust and could be induced by synthetic odors (Endevelt-Shapira et al. 2018). This hints to a disrupted processing of olfactory stimuli, especially of social odor cues in ASD, whereby the subliminal effects of effects of social stimuli on autonomic circuitry and function are abnormally processed. Hence, a component of the stimulus origin and setting plays a crucial role in impairment selectivity for social as opposed to non-social odor processing.

### Autism candidate genes in olfactory circuit wiring

Throughout life, the olfactory system is a highly malleable and plastic network due to the continuing integration of adult-born neurons and activity-dependent synaptic plasticity. However, olfactory circuit assembly during prenatal and early development is innately determined by genetic factors and regulated by a variety of transcription factors. Genetic factors dynamically influence not only circuit assembly but also synaptic connectivity. Transcriptional control of the main olfactory bulb (MOB) circuit assembly and its topographic organization, for one, has been reviewed previously (Sakano 2020). However, here the focus is on the transcriptional control of cortico-bulbar connectivity due to its potentially greater implication in ASD, although much less is known about this part of olfactory circuit development.

As a spectrum disorder, varying phenotypes may be expressed across individual ASD cases as subject to the underlying genetic mutation, and the same phenotypes can be expressed by different genetic mutations. Environmental factors, such as upbringing, additionally influence the development of autistic phenotypes and affect sensory information processing and valuation. Undoubtedly, the causes of ASD are multi-faceted and should be considered from a holistic perspective which bridges genetics and behavior.

The rodent olfactory system processes volatile and non-volatile stimuli with both playing a role in social odor processing. Non-volatile components, such as steroid derivatives in urine, are primarily processed by the accessory olfactory system (AOS) (Baum and Kelliher 2009; Liberles 2014). The primary sensory neurons of the AOS are located in the vomeronasal organ (VNO) at the base of the nasal septum. They detect stimuli by a class of G-protein coupled receptors, which differ from olfactory sensory neurons (OSNs) in the olfactory epithelium. VNO sensory neurons extend their axons to the glomeruli of the accessory olfactory bulb (AOB), which is an adjacent, but separate structure to the MOB. AOB outputs predominantly bypass the olfactory cortex and project directly to the medial nucleus of the amygdala (MeA) and the posterior bed nucleus of the stria terminalis (BNSTp) (Holy 2018).

Volatile components of odors are mostly sampled and processed by the main olfactory system (MOS). The neural circuit of the MOS starts with the OSNs in the olfactory epithelium projecting to the glomeruli of the MOB, essentially forming a topographic map (Mombaerts et al. 1996; Mombaerts 2006; Imai and Sakano 2007; Takeuchi et al. 2010; Wu et al. 2018). Mitral cells and middle tufted cells (MC/MTC) in the MOB then project to primary olfactory regions, such as the piriform cortex and the anterior olfactory nucleus, as well as to the olfactory tubercle of the ventral striatum and other limbic regions. Within the MOB, MC/MTCs extend lateral dendrites, forming dendro-dendritic synapses with inhibitory granule cells, to elicit lateral inhibition of other MC/MTCs involved in local oscillatory activity and odor discrimination (Margrie et al. 2001; Lepousez et al. 2014; Kollo et al. 2014). Inhibitory granule cells are located at the center of the MOB and form the largest cell population. The abundant top-down influence from olfactory cortices to the MOB mainly targets the granule cells (for a review of the MOS, see Brann and Datta 2020; Schäfer et al. this issue). Top-down influence on MOB by the AON and the anterior piriform cortex has been described previously (Boyd et al. 2012, 2015; Markopoulos et al. 2012; Rothermel and Wachowiak 2014; Oettl et al. 2016; Quintela et al. 2020 and reviewed by Rothermel et al. this issue**)**.

Various genetic factors dynamically influence the assembly and refinement of cortico-bulbar synaptic connectivity. However, little is known about their functional implications in coding (Ravi et al. 2017). Future studies warrant investigation of the influences of olfactory coding genes on neuronal population activity and behavioral output.

To further understand the physiological signatures of disease-related phenotypes, the underlying neuronal circuitry can be examined. Previous research on ASD, largely focused on microcircuitry dysfunction (Rinaldi et al. 2008), suggested that ASD-related perturbations of neuronal circuit wiring disrupt the balance of excitatory and inhibitory activity (E/I balance). This imbalance has been implicated in a variety of other neuropsychiatric disorders and diseases, for instance, in stress disorders (Rosenkranz et al. 2010; McKlveen et al. 2019), schizophrenia, and epilepsy (Marín 2012; Selten et al. 2018).

Genetic deficits in neuropsychiatric and neurodevelopmental disorders with disturbed E/I balance and synaptic weighting (Selten et al. 2018) also influence sensory processing and brain function at the systems-level. Several ASD candidate genes have been identified throughout the last two decades. A significant number of these genes encode proteins involved in the formation and maintenance of synaptic contacts, including *Cntnap2*, *Nlgn3*, *Shank2*, *Shank3*, and *Tbr1* (see also Table 1), to name some of the most relevant (Huang et al. 2014; Bourgeron 2015; De Rubeis and Buxbaum 2015; de la Torre-Ubieta et al. 2016). The functional consequences of altered synaptic development and the function of autism candidate genes are highlighted here using an example of selected *Shank*-family members.

**Table 1:** Genetic knockout and inbred ASD mouse models with altered chemosensory functions. The table provides an exemplary list of common rodent models and their association to ASD, including behavioral and olfactory phenotypes as well as successful rescue experiments

Gene	Relation to ASD	Mouse phenotype	Olfactory function	Rescue	References
*Cntnap2* (also known as *Caspr2* encodes a transmembrane protein member of the neurexin family)	Identified as a risk gene, especially in families where all affected children were male (Alarcón et al. 2008; Arking et al. 2008)	Hyperactivity, epileptic seizures, impaired social interaction, vocal communication and repetitive behaviors (Peñagarikano et al. 2011)	Intact olfactory function (Levy et al. 2019) or even better performance than wild type mice in the buried food test (Peñagarikano et al. 2011)	Risperidone administration can rescue hyperactivity and motor stereotypies, while OXT can rescue the social impairments (Peñagarikano et al. 2011)	Alarcón et al. (2008), Arking et al. (2008), Ma et al. (2009); Peñagarikano et al. (2011), and Levy et al. (2019)
*Grin1* (NMDAR subunit, also known as NR1 or NMDAR1; originally proposed as a model for schizophrenia)	A range of neuropsychiatric disabilities that share autistic features and intellectual disability have been described (Rossi et al. 2017)	Knockdown model: hyperactivity, stereotyped behavior, impaired social behavior (Mohn et al. 1999)	Overall intact olfaction, but some behavioral differences in olfactory-based tasks have been described (Duncan et al. 2004; Moy et al. 2008a)	Knockdown model: Oxytocin increases sociability (Teng et al 2016)	Mohn et al. (1999), Duncan et al. (2004), Moy et al. (2008a), Teng et al. (2016), and Rossi et al. (2017)
*Nlgn3* (Neuroligin3 is a neuronal cell surface protein involved in synaptic formation and remodeling)	Mutations and de novo copy number variations on the X-linked gene *Nlgn3* were found in ASD patients (Jamain et al. 2003; Gilman et al. 2011; Sanders et al. 2011)	Hyperactivity, motor stereotypies, and impaired social behavior (Rothwell et al. 2014)	Lower performance in buried food test, possibly reflecting olfactory impairment (Radyushkin et al. 2009)	Rescue of social novelty response by re-expression of *Nlgn3* in dopaminergic cells in juvenile mice or restoration of oxytocin responses through a modulator of translational homeostasis (Hörnberg et al. 2020)	Jamain et al. (2003), Radyushkin et al. (2009), Gilman et al. (2011), Sanders et al. (2011), and Rothwell et al. (2014)
*Oxtr* (Gq-coupled receptor for the neuropeptide oxytocin)	Association of *Oxtr* single nucleotide polymorphisms with ASD diagnosis found in Asian and Caucasian samples (Wu et al. 2005; Jacob et al. 2007)	Impaired social behavior and communication (Takayanagi et al. 2005; Pobbe et al. 2012) as well as reduced cognitive flexibility and higher susceptibility to seizures (Sala et al. 2011)	Abnormal olfactory behavior seen in an olfactory avoidance task (Osada et al. 2018)	Intracerebroventricular administration of OXT or vasopressin can rescue social behavior, impaired cognitive flexibility, increased aggression and seizure susceptibility (Sala et al. 2011)	Wu et al. (2005), Takayanagi et al. (2005), Jacob et al. (2007), Sala et al. (2011), Pobbe et al. (2012), and Osada et al. (2018)
*Shank2* (a member of the Shank family scaffolding proteins found in excitatory synapses)	de novo copy number variations found in ASD patients (Berkel et al. 2010; Leblond et al. 2012)	Stereotypical behavior, hyperactivity, increased anxiety and impaired social behavior (Schmeisser et al. 2012; Won et al., 2012)	Altered E/I in olfactory cortex of *Shank2* overexpressing mice (Eltokhi et al., unpublished data)	Modulation of NMDAR function (e.g. with D-Cycloserine) improved social function (Won et al. 2012)	Berkel et al. (2010), Leblond et al. (2012), Schmeisser et al. (2012), and Won et al. (2012)
*Shank3*	Haploinsufficiency is the most probable cause of the 22q13 deletion syndrome (Phelan–McDermid syndrome) and further variations/mutations of Shank3 have been associated with ASD (Durand et al. 2007; Moessner et al. 2007; Gauthier et al. 2009)	Repetitive behavior, impaired social behavior (Peça et al. 2011)	Normal olfactory habituation/dishabituation in *Shank3*-deficient mice and rats (Bozdagi et al. 2010; Song et al. 2019)	Rescue of social behavior by various interventions, e.g. adult restoration of Shank3 expression (also repetitive behavior) (Mei et al. 2016) or oxytocin treatment (Harony-Nicolas et al. 2017)	Durand et al. (2007), Moessner et al. (2007), Gauthier et al. (2009), Bozdagi et al. (2010), Peça et al. (2011), Mei et al. (2016), HaronyNicolas et al. (2017), Song et al. (2019)
*Tbr1* (transcription factor involved in cortical development)	Sequencing studies in ASD patients found reoccurring de novo mutations of Tbr1 (O’Roak et al. 2012; Deriziotis et al. 2014)	Tbr1 -/-: neonatal lethality (Hevner et al. 2001) Tbr1 -/+ : impaired cognitive flexibility, associative memory and social interaction (Huang et al. 2014)	Tbr1-/-: loss of most projection cells in the MOB, impairment in neuronal migration (Bulfone et al. 1998)	Tbr1 -/+ : NMDA-R agonists (D-Cycloserine or Clioquinol) can rescue behavioral phenotype (Huang et al. 2014; Lee et al. 2015)	Bulfone et al. (1998), Hevner et al. (2001), O’Roak et al. (2012), Huang et al. (2014), Deriziotis et al. (2014), and Lee et al. (2015)
*C58/J* Inbred mouse strain	Genetic association not known	Repetitive behaviors, impaired social behavior in males (social approach, social transmission of food preference) (Moy et al. 2008b)	Basic olfactory screening normal (Ryan et al. 2010)	Oxytocin increases sociability (Teng et al. 2016) and other pharmacological interventions rescue repetitive behavior (Silverman et al. 2015)	Moy et al. (2008b), Ryan et al. (2010), Silverman et al. (2015), and Teng et al. (2016)
*BTBR T* + * Itpr3tf/J* Inbred mouse strain	Several ASD-relevant genetic variations have been identified as compared with C57BL/6 (McFarlane et al. 2008; Daimon et al. 2015)	Impaired social behavior, social transmission of food preference and repetitive behaviors (McFarlane et al. 2008)	Basic olfactory screening normal, but taste dysfunction (Tordoff and Ellis 2013)	Various pharmacological interventions have shown rescue of social impairments (for review see Meyza and Blanchard 2017)	McFarlane et al. (2008), Tordoff and Ellis, (2013), Daimon et al. (2015), and Meyza and Blanchard, (2017)

Numerous studies have suggested that Shank impairment is involved in the development of ASD (Grabrucker et al. 2011; Jiang and Ehlers 2013; Guilmatre et al. 2014; Monteiro and Feng 2017). Shank proteins comprising Shank1, Shank2, and Shank3 are associated with postsynaptic scaffolding and play a critical role in structuring excitatory synapses (Schmeisser 2015; Ey et al. 2020). Under-connectivity, as defined by the reduced growth of dendrites, axons, and synapses (Fernandez et al. 2019), may be reflected at the systems-level, and characterized by reduced, or aberrant, inter-regional connectivity. Such pathogenic features have been illustrated, for instance, by *Shank3* deficiency; whereby, functional connectivity was affected as well as the structure of prefrontal regions (Pagani et al. 2019). At the molecular level, Schmeisser and colleagues (2012) found that both *Shank2* and *Shank3* mutant mice displayed altered synaptic expression of receptors specific to excitatory synaptic transmission in various brain regions. Furthermore, depending on the localization of *Shank2*-associated neuronal deficits, different ASD phenotypes can be observed (Kim et al. 2018). For instance, *Shank2* deletion in parvalbumin-positive neurons leads to moderate hyperactivity and enhanced self-grooming (Lee et al. 2018), while *Shank2* deficiency in cerebellar Purkinje cells has been linked to impaired social interactions as well as motor coordination deficits (Peter et al. 2016). Importantly, data also indicate that Shank2 deficiency in the hippocampus and striatum contribute critically to the phenotypic impairments in social and motor behaviors (Schmeisser et al. 2012; Won et al. 2012). Overall, in mouse models of ASD, sensory, prefrontal, hippocampal, cerebellar, and striatal regions, as well as the circuits that connect them are perturbed (Golden et al. 2018). These circuits differentially contribute to ASD-related behaviors, a pattern that has emerged across several major ASD mutant mouse lines (Rothwell et al. 2014; Bey et al. 2018; Zerbi et al. 2018; Guo et al. 2019).

### Olfactory coding of social cues

Thus far, the importance of olfaction in guiding social behavior not only in rodents but also in humans was illustrated. We further described the influence of genetics on neuronal assemblies and synaptic wiring in the olfactory system. Next, is to address the question of how social odor information is typically processed. The circuitry supporting social odor processing occupies several brain regions, and these sub-regions form distinct, parallel functional pathways, as evidenced by human fMRI (Zhou et al. 2019).

Intact social behavior is reliant upon both the MOS and the AOS. Investigating immediate early gene (*c-Fos*) expression in the male brain after interaction with a same- or other-sex conspecific, or with a non-social odor, showed a large overlap in the activation of MOS-associated regions, namely, the AON, piriform cortex, cortical amygdala (CoA), and the lateral entorhinal cortex (Kim et al. 2015). AOS-associated brain regions like the MeA and parts of the CoA showed higher *c-Fos* expression for opposite sex stimuli. Of note, male interaction with female conspecifics showed specific activation of the tenia tecta and post-piriform transition area and stronger recruitment of the MOS-pathway-associated ventral striatum and orbitofrontal cortex (Kim et al. 2015).

In a functional magnetic resonance imaging (fMRI) study, both the MOS and AOS pathways were activated after the presentation of volatile urine odors and pheromones with different selectivity (Xu et al. 2005). After lesioning of the main olfactory epithelium, mice lost the ability to distinguish between volatile and non-volatile opposite-sex olfactory cues (Keller et al. 2006). In contrast, the destruction of the VNO resulted in no impairment of sex discrimination and recognition of volatile and non-volatile odor components (Pankevich et al. 2004). Mutant studies with impaired action potential generation in the VNO sensory neurons showed reduced aggressive behavior and increased mounting behavior towards a male intruder (Clancy et al. 1984; Leypold et al. 2002). This further implicates the AOS in aggression and sexual behavior, but not for individual recognition (Chamero et al. 2007, 2011).

Most social behavior, typically associated with the MOS, needs to be flexible and context-dependent. Two chemically identical social odors can elicit different behavioral responses based on the internal state of the animal. This internal state can contain learned associations and values of an odor, as well as physiological latent variables like arousal, attention, and environmental context (Chen and Hong 2018). For example, after associative learning, mice showed preference to a previously neutral odor after conditioning the odor with a social cue (Choe et al. 2015).

Moreover, an example of social transmission, as seen with food preference, is where mice favor one source of food over another if they have smelled it on the breath of a demonstrator animal before (Munger et al. 2010; Wang et al. 2020). The acquisition of socially transmitted food preferences in this case was mediated by specialized OSNs, expressing a specific receptor unit, namely, guanylyl cyclase, which binds breath odorants. The circuitry processing social odor information is, therefore, selective to stimuli at the initial level of odorant binding. However, even with this selectivity, the processing of social odor information cannot simply be split into “social” and “non-social,” without also considering how sensory processing is modulated by one’s internal state (e.g., containing previous experiences, recognition) in a contextual manner to correctly extract the social information (Chen and Hong 2018).

Social behavior requires state- and context-dependent extraction and evaluation of social information from a rich multisensory input consisting of both social and non-social, relevant and irrelevant features. Examining distributed circuitry has revealed, in humans, the involvement of not only primary sensory processing cortices but also higher-order brain regions in appetitive and aversive olfactory learning (Gottfried et al. 2002), in encoding predictive reward value (Gottfried et al. 2003), and in odor percept formation (Li et al. 2008). With this methodological approach, the presentation of social reinforcement learning paradigms can help measure the values assigned to social odors across multiple regions.

Decoding these responses facilitates the interpretation of affective and value encoding. Social odor learning and memory is capable of modulating innate, endogenous responses to a pure sensory stimulus, coupling such processing of incoming stimuli with a learned preference and/or valence (Macrides et al. 1982; Gottfried et al. 2002). Odor-discrimination learning is supported by the hippocampus and relies on both higher-order and primary sensory cortices (Martin et al. 2007). Higher-order areas, such as the ventral hippocampus, exhibit direct projections to the AON, whereby top-down input by the hippocampus on AON modulates the processing of incoming olfactory information, especially in regard to contextual processing and stored internal representations (Aqrabawi and Kim 2018; Levinson et al. 2020). In this manner, the AON may act as a central hub between olfactory input and odor memory (Oettl et al. 2016; Linster and Kelsch 2019; Aqrabawi and Kim 2020). To this effect, the AON is necessary for the expression of behavior relevant to the social odor memory.

All in all, both external input and internal representations contribute to dynamic changes in network processing. The network for the processing of social cues encompasses distributed circuits across primary sensory and higher-level cognitive areas that interact selectively to produce flexible social behavior.

### Neural processing of social odors in ASD models and patients

Studies have examined the underlying neuronal networks afflicted by genetic mutations responsible for ASD characterization. Indeed, affected neuronal networks have resulted in impaired processing across multiple sensory input domains (Thye et al. 2018). However, here the focus is placed on olfactory decoding of social odors. Olfactory-based task impairment has been noted in many ASD animal models (Silverman et al. 2010; Won et al. 2012). Yet, similar to ASD patients, the olfactory detection threshold has been tested in some genetic mouse models and appeared preserved (Peñagarikano et al. 2011; Won et al. 2012; Levy et al. 2019; see also Table 1).

A reoccurring finding in many animal models that exhibit behavioral impairments in the social domain is a disrupted E/I balance in a group of locally connected neurons (i.e., the microcircuit) (Lee et al. 2017). This could be a comprehensive link for bridging the level of single neurons and synaptic development to the whole system and to help understand the impact of altered synaptic wiring and circuit assembly by genetic causes. It is sufficient in the prefrontal cortex (PFC) to acutely increase E/I balance in a typically-developed rodent brain to induce altered social behavior (Yizhar et al. 2011). However, the underlying mechanism causing the E/I imbalance in distributed circuits is most likely rooted in genetic control of processes at the circuit and synapse levels (see Lee et al. 2017 for comprehensive review of ASD candidate genes and E/I balance). E/I imbalance in ASD models has been observed in prefrontal circuits (Levy et al. 2019), where disrupted E/I in *Cntnap2* knock-out mice was reflected by higher noise levels in spontaneous population activity. However, other examples of E/I imbalance in other brain regions (i.e., amygdala; Lin et al. 2013) support a more systemic role of E/I balance that may influence multiple functional circuits.

Functional impairments exhibited by changes in typical olfactory wiring can affect odor discrimination and perception. Such functional processing relies heavily on the anterior piriform cortex and is facilitated by its connections to the CoA and the posterior piriform cortex, which is reciprocally connected with thalamo-cortical circuitry and higher-order cortical domains, such as the orbitofrontal cortex (Wilson et al. 2006). In a previous study measuring behavioral and fMRI responses, researchers found that ASD patients had lower odor identification scores as compared with healthy controls and exhibited decreased BOLD signal in the anterior piriform cortex during odor stimulus processing (Koehler et al. 2018). Whole-brain studies employing fMRI, either at rest or during social stimulus processing, can provide a framework for understanding ASD impairments at the systems-level and the implication for canonical networks, such as the saliency network (Uddin and Menon 2009), in social odor processing.

Recent unpublished work (Eltokhi, Oettl, Rozov, Kelsch, Rappold, Sprengel, unpublished observations) examined the functional consequences of manipulating the expression of wild-type and human point mutations in ASD cases of the *Shank2a* gene and addressed olfactory coding in the AON. They observed that transgenic overexpression of the wild-type *Shank2a* gene in glutamatergic forebrain neurons decreased social exploration, while expression of a human point mutation of *Shank2a* led to higher levels of conspecific exploration than in matched controls. These social behavior phenotypes were reversed when overexpression of the genes was switched off in the adult, suggesting that contrary to other behavioral alterations that persisted, are not hard-wired neurodevelopmental processes, but accessible to adult causal interventions. These two models of *Shank2a* dysfunction displayed altered proteomic expressions of synaptic wiring proteins and bi-directional changes in their synaptic functions. Also, the network activity in the AON was modified in the two transgenic models. Specifically, in awake mice, background network activity was altered, and the E/I ratio of stimulus responses was changed bi-directionally. An excess of wild-type *Shank2a* displayed decreased social exploration and an enhanced excitatory baseline drive with more background noise in the AON, while the point mutant model with increased social approach behavior displayed an increased sparseness in AON activity with lower E/I balance than controls and higher signal-to-noise in cortical odor stimulus coding.

With ASD rodent models, genetic re-expression, optogenetic, and pharmacological techniques may be employed in attempts to rescue disrupted behavioral phenotypes. For instance, rescue of social impairments was also accomplished in a *Shank3* knock-out murine model via genetic re-expression of the deleted gene (Mei et al. 2016). The evidence for the role of *Shank* genes in ASD is growing along with attempts at selectively targeting genes for therapeutic treatment. This hints that the changes induced by genetic alterations are not completely hard-wired, but can be rescued by normalizing the expression of the gene that caused the alterations in social behavior (Mei et al. 2016).

Pharmacological treatments targeting the underlying molecular pathways have shown some success in mice, whereby inhibition of the ERK signaling pathway resulted in rescuing the pathophysiology and behavioral phenotype associated with 16p11.2 chromosomal deletion (Pucilowska et al. 2018). Pharmacological intervention in human cases may also be possible. For instance, with the pro-social neuro-hormone oxytocin that drives social stimulus processing in the brain, but its release is reduced in autism models (Peñagarikano et al. 2015; Sgritta et al. 2019; Resendez et al. 2020), and potentially also in human ASD cases (Husarova et al. 2016; Rutigliano et al. 2016; Zhang et al. 2016).

**Fig. 2:**
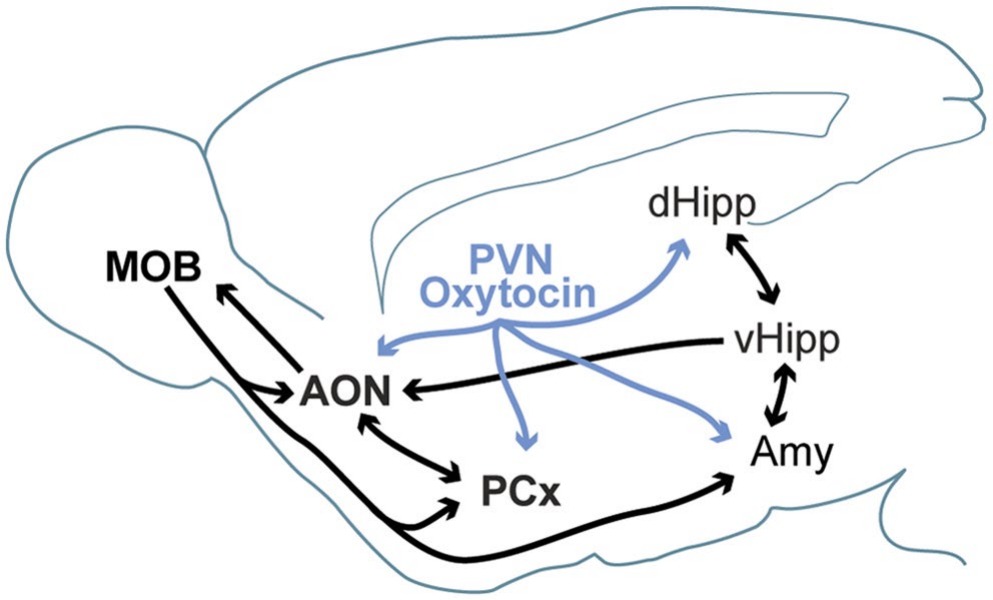
Primary circuit wiring for olfaction and oxytocin. The primary olfactory processing circuitry is depicted alongside oxytocin projection sites. Incoming stimuli (e.g., social odorants) are first processed by the MOB before relay to the AON, PCx, and the Amy. Recurrent projections between bottom-up and top-down processing streams are illustrated by the double-sided arrows. The central hub for oxytocin production, the PVN, is shown projecting to downstream effector targets that can have a direct or indirect effect on olfactory processing. Abbreviations: AMY amygdala; AON anterior olfactory nucleus; dHipp and vHipp the dorsal and ventral hippocampus, respectively; MOB: main olfactory bulb; PCx piriform cortex; PVN paraventricular nucleus of the hypothalamus

Oxytocin is produced in the paraventricular nucleus (PVN) of the hypothalamus and released by neurons projecting to several brain regions, including primary olfactory regions (Yu et al. 1996; Choe et al. 2015; Oettl et al. 2016), the cortical amygdala (Knobloch et al. 2012), and the dorsal hippocampus (Tirko et al. 2018) (see Fig. 2). There is a tight relationship between the oxytocin and reward pathways (Dölen et al. 2013; Hung et al. 2017; Walum and Young 2018), whereby this interplay can greatly influence social behavior, reward, and value assignment. Oxytocin has been shown to influence E/I balance in higher-order brain regions, such as the hippocampus (Owen et al. 2013) and also to modify the signal-to-noise ratio for odors in the MOB through increased top-down inputs from the AON (Oettl et al. 2016; Oettl and Kelsch 2018). Electrophysiological recordings of oxytocin neurons in rats have shown activation during social interaction in an open field and selective manipulation of oxytocinergic activity, using a chemogenetic intervention, controlled motivation for social behavior (Tang et al. 2020). A different study using in-vivo 2-photon microscopy in head-fixed mice exhibited increased activity of oxytocin neurons to a social odor, specifically that of a juvenile animal (Resendez et al. 2020).

However, an immunohistological investigation revealed significantly lower numbers of immunopositive oxytocin cells in the paraventricular nucleus of the hypothalamus (PVN) in *Shank3b* and *Cntnap2* mutant mice (Peñagarikano et al. 2015; Sgritta et al. 2019; Resendez et al. 2020). Could oxytocin (or related neuromodulators), therefore, act as social boosters to enhance or restore social information perception, and potentially serve as an ASD treatment (Young and Barrett 2015)?. This may be possible since bi-directionally modifying oxytocin release and actions in the AON resulted respectively in gain and loss of function in social exploration and memory formation of conspecifics (Oettl et al. 2016).

This question was addressed in animal models of ASD by performing rescue experiments, where oxytocin was administered to see if it would restore the healthy behavioral phenotype. *Shank3b* knockout mice showed decreased sociability in a 3-chamber test (Peça et al. 2011), but the social deficits could be reversed by acute intraperitoneal injection of an oxytocin receptor agonist (Resendez et al. 2020). Intracerebroventricular infusion of oxytocin in a *Shank3* deficient rat model reversed the deficit in long-term social memory and also improved attention in a non-social task (Harony-Nicolas et al. 2017).

In *Cntnap2* knockout mice, acute oxytocin release using a chemo-genetic intervention showed rescue of social deficits in the sociability and reciprocal social interaction tests (Peñagarikano et al. 2015). Further, studies using two different models for ASD, *C58/J* and *Grin1*, showed increased sociability for up to 2 weeks after sub-chronic oxytocin treatment (Teng et al. 2016). A different study investigating the effect of long-term exposure to intranasally administered oxytocin in another mouse model of ASD (*BTBR T*+ *Itpr3tf/J* inbred strain) only found increased female sniffing in the 3-chamber test, but no effect on other ASD-related behavior (Bales et al. 2014). Oxytocin also mediates the influence of the microbiome on social behaviors (Sherwin et al. 2019). Ingestion of Lactobacillus reuteri, a bacterial species found in the gut system (Britton 2017), leads to a behavioral rescue across a variety of genetic, environmental, and idiopathic ASD models (Sgritta et al. 2019). The change in social behavior was dependent on an intact vagus nerve and absent in animals lacking the oxytocin receptor in dopaminergic neurons of the ventral tegmental area (Sgritta et al. 2019). Also, restoration of oxytocin-signaling in the dopaminergic neurons rescued social novelty responses in *Nlgn3*-mice (Hörnberg et al. 2020). These examples from animal experiments show that oxytocin can increase sociability and social cognition across different genetic ASD models. In parallel to the observed disruption of the E/I balance in mouse models of ASD, oxytocin modifies the E/I ratio across sensory and higher-order brain circuits to influence social behavior.

## Future directions

A further examination of disrupted brain networks in ASD is required to fully understand the underlying patho-mechanisms. While a finite number of appropriate tests exists, it is nonetheless of principle importance that behavioral deficits are properly evaluated to the extent warranted. This calls for future studies employing multi-modal methodology (i.e., molecular, cellular, and systems-wide) to unravel impaired behavioral phenotypes throughout the biological framework. Ascribing behavioral phenotypes to specific genes may further help to predict and localize deficits.

Neuronal activity underlying social odor processing may be captured using multi-site extracellular recordings, where the coherence of activity between stimulus processing hubs can be determined. Such data are rich in the sense of temporal resolution and could be complemented with a whole-brain approach (e.g., fMRI) for systems-level decoding of olfactory processing. Prior human fMRI findings, for example, could serve as a reference for executing similar experiments in animals where in vivo cell recording is also permitted, incorporating another facet of data collection. Moreover, the development of promising non-human primate models for ASD (Bauman and Schumann 2018; Tu et al. 2019) may contribute to translating a repertoire of animal research to humans.

Considerations of both brain and body states should be taken into account in order to appropriately decode neuronal responses to social cues. Concurrent measurements of physiological parameters may help elucidate state-specific responses during social information processing. Insofar, physiological parameters may serve as another therapeutic target (Garfinkel et al. 2016). Here, clinical intervention can incorporate focused breathing and interoceptive feedback into patient care (Schauder et al. 2015).

Further, exploiting the inter-connectedness of oxytocin with the olfactory and reward learning systems may assist with typical processing of salient social stimuli. Notably, social memories affect odor discrimination and identification as well as the contextual cues and associations constituting odor percepts. Therefore, consideration of the recurrency between higher-order processing hubs and primary olfactory processing regions is vital to understanding the extent of ASD patho-mechanisms and their effects at the systems-level.

## Conclusion

In conclusion, olfactory processing influences social behaviors profoundly in rodents and in humans. Therefore, it is to no surprise that ASD also displays impairments in the olfactory system. Here, we compared social odor processing in healthy individuals and in ASD and described some of the current known influences of ASD-related genes on functional encoding in the social sensory domain. The current relatively small number of neurophysiology studies suggest alterations in the E/I ratio as a recurring signature in genetic disease models and is shared in primary sensory areas and not limited to higher-order cortices. This resonates well with the finding that ASD also shows impairments in early sensory perception as well as higher cognitive domains. A large number of the identified risk genes for ASD play a role in the assembly and maintenance of synapses. The disrupted E/I balance observed in ASD models might therefore be an indirect reflection of the genetic impairment of synaptic organization. Yet, other more specific alterations in coding need to be identified since the increased E/I signature is also found in other psychiatric disorders like schizophrenia or depression.

In light of the need for developing therapeutic targets in ASD, genetic, pharmacological, and technical manipulations emerge as entry points for ASD phenotype rescue. Hope for novel treatment options also lies in the findings of recent studies that have achieved phenotype rescue by restoring correct genetic expression of autism candidate genes, such as members of the Shank family in adult rodents. Further, the potential role of neuro-hormones, such as oxytocin, in ameliorating impaired social olfactory processing was highlighted as one potential therapeutic target. Because oxytocin has also been shown to modulate E/I ratio and signal-to-noise across brain areas, it might further provide a comprehensive link to better understand social processing in health and patients.

## Abbreviations

AOS: Accessory olfactory system
AON: Anterior olfactory nucleus
ASD: Autism spectrum disorder
E/I: Excitation to inhibition balance
MC: Mitral cell
MOS: Main olfactory system
MTC: Middle tufted cell
OSN: Olfactory sensory neuron
PCx: Piriform cortex
